# Liver and Renal Injury with Remdesivir Treatment in SARS-CoV-2 Patients

**DOI:** 10.12669/pjms.39.2.6236

**Published:** 2023

**Authors:** Rabiah Sadaf, Faiza Sadaqat Ali, Tazeen Rasheed, Bader Faiyaz Zuberi

**Affiliations:** 1 Rabiah Sadaf, FCPS., Consultant Physician, Dr Ruth K.M. Pfau, Civil Hospital Karachi, Pakistan; 2 Faiza Sadaqat Ali, FCPS., Senior Registrar, Department of Medicine, Dow Medical College, Dow University of Health Sciences, Karachi, Pakistan; 3 Tazeen Rasheed, FCPS., Associate Professor, Department of Medicine, Dow Medical College, Dow University of Health Sciences, Karachi, Pakistan; 4 Bader Faiyaz Zuberi, FCPS., Meritorious Professor, Clinical Trials Unit, Dow University of Health Sciences, Karachi, Pakistan

**Keywords:** Creatinine clearance, Liver function tests, SARS-CoV-2, Remdesivir

## Abstract

**Objective:**

To determine the effect of Remdesivir on liver enzymes and renal functions in SARS-CoV-2 patients.

**Methods:**

This prospective cohort study was conducted at Dr. Ruth KM Pfau, Civil Hospital Karachi between 1^st^ December 2021 to 31^st^ January, 2022. All patients of severe SARS-CoV-2 infection who received Inj. Remdesivir for five days as per protocol of SARS-CoV-2 management were included. Biodata of selected patients including age, gender, diabetic, hypertensive status was recorded. Patients Liver Function Tests and Serum Creatinine were performed on days 0, 3, 5, 7 and 14.

**Result:**

This study included 85 patients, out of which 55 (64.7%) were males and 30 (35.3%) were females. Out of 85 patients, Remdesivir was stopped in 3 (3.5%) patients. Among these three patients Remdesivir was stopped in one patient on day three because of decrease in CrCl to <30 ml/min. His CrCl improved after stopping Remdesivir. In the remaining two patients, Remdesivir was stopped due to increase in ALT to greater than 10 times from normal values on day three. Similarly, in these two patients the ALT improved after stopping Remdesivir.

**Conclusion:**

Only three patients developed adverse effects resulting in stopping of Remdesivir, however these were reversible on stopping the drug. Therefore, Remdesivir is a relatively safe drug and well tolerated in SARS-CoV-2 patients.

## INTRODUCTION

Since the emergence of highly contagious respiratory illness caused by SARS-CoV-2 virus in December, 2019 from Wuhan, China SARS-CoV-2 has become a global pandemic affecting almost every aspect of life. This pandemic has devastated economies and given surpassing challenges to the healthcare systems around the world.^1^ To counteract this threat several drugs have been investigated, one of them is Remdesivir which got FDA approval in 2020 for the treatment of Corona virus disease.^2^

Remdesivir is an antiviral which was first developed in 2016 to control Ebola Virus disease outbreak in Africa.^3^ Several in vivo and in vitro studies in animal models suggested its potential broad spectrum activity against other viruses as well such as SARS, MERS, Marburg and SARS-CoV-2.^4^ Remdesivir and its active metabolite are mostly (74%) eliminated by kidneys. The plasma half-life of the parent drug is short around one to two hours, but the half-life of its active metabolite Remdesivir triphosphate is about 20–25 hours. Remdesivir has limited water solubility, largely excreted through glomerular filtration, hence its elimination becomes slow in patients with abnormal renal functions. In the liver, Remdesivir is metabolized by CYP3A4.^5^

There is limited local data regarding the hepatic and renal safety profile of this drug in our population, making treatment decisions difficult for physicians, therefore we planned this study to assess the effects of this only approved antiviral drug till date on liver and renal profile in our population. This may guide how frequently liver enzymes and serum creatinine should be monitored in SARS-CoV-2 patients taking Remdesivir and when the drug should be stopped. The objective was to determine the effect of Remdesivir on liver enzymes and renal functions in SARS-CoV-2 patients.

### Operational definitions:

### Severe SARS-CoV-2 Infection:

Individuals with SpO_2_ < 94% on room air, respiratory rate of > 25 breaths/min, or lung infiltrates on CXR > 50%, requiring supplemental oxygen.^6^

### Normal values of Liver Function Tests:

ALT: 19-25 IU/L for females; 29-33 IU/L for males^7^

### Alkaline phosphatase:

50-100 U/L, Total bilirubin: 0.3-1.0 mg/dl

*Normal value of CrCl:* Male: 110 to 150 ml/min, Female: 100 to 130 ml/min^8^

### Creatinine Clearance (mL/min):

“calculated using Cockcroft-Gault formula”^9^


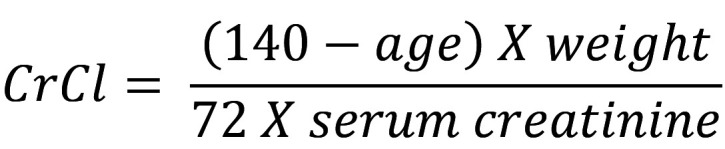


For females result of the equation was multiplied by 0.85.

(Age in years; weight in Kg; Serum Creatinine in mg/dl)

## METHODS

This Prospective Cohort study was conducted at Dr. Ruth KM Pfau, Civil Hospital Karachi between 1^st^ December,2021 to 31^st^ January,2022 satisfying inclusion/exclusion criteria were included after informed consent and ethical approval (IRB-2268/DUHS/Approval/2021/619 dated 29^th^ November, 2021) was taken from Institutional Review Board of Dow University of Health Sciences. A total of 85 patients were included. Non-probability consecutive sampling was used for selection of patients.

### Inclusion criteria:

All patients of severe SARS-CoV-2 infection who received Inj. Remdesivir for 5 days as per protocol of SARS-CoV-2 management.^11^

### Exclusion criteria:

Patients having ALT > 5 times the ULN prior giving Remdesivir, known patients of HBV, HDV & HCV, patients having eGFR < 30 ml/min, pregnant or breast-feeding females, patients with known hypersensitivity to Remdesivir, those who expired before 14^th^ day when last sample was collected were excluded.

Given frequencies from previous study of liver 35% involvements in SARS-CoV-2 patients receiving Remdesivir. Using power of 80.0% to detect difference of P0-P1 of -0.15 and alpha of 0.05, the sample size was calculated as 85. Calculation was done using PASS software.^12^ All admitted patients meeting inclusion criteria were included after taking informed consent by non-parametric consecutive sampling method. Biodata of selected patients including age, gender, diabetic, hypertensive status was recorded. After all aseptic measures blood was drawn by a trained phlebotomist for Liver Function Tests and Serum Creatinine on days 0, 3, 5, 7 and 14. On day zero tests for HBs Ag and HCV Ab were also done. LFTs and serum creatinine were done by photometric method using Cobas C501 analyzer.

### Data analysis:

Frequencies of gender, diabetes and hypertension were reported and compared by χ^2^-test. Frequencies of different adverse effects on liver and renal functions were reported. Means ±SD of quantitative variables like age, duration, liver enzymes values and renal parameters were determined and compared by Student’s t-test. Significant level was set at ≤.05. Data was analyzed using SPSS version 26.

## RESULTS

In this study eighty-five patients admitted in SARS-CoV-2 treatment facility of Dr Ruth KM Pfau, Civil Hospital Karachi were enrolled. Mean ±SD of age of patients was 51.47 ±13.39. Out of the 85 patients 55 (64.7%) were males and 30 (35.3%) were females. Details of frequencies of Hypertension, Diabetes Mellitus and age and their statistical comparison is given in Table-II. The Mean ± SD of CrCl & ALT on days 0, 3, 7 & 14 are shown in Table-III.

**Table-I:** DIADS table for grading the severity of adverse events^10^

Grades	1	2	3	4
ALT/Alkaline Phosphatase	1.25 to <2.5 x ULN	2.5 to <5.0 x ULN	5.0 to <10.0 x ULN	≥10.0 x ULN
Direct Bilirubin	N/A	N/A	>ULN with other signs and symptoms of hepatotoxicity.	>ULN with life-threatening consequences (e.g., signs and symptoms of liver failure)
CrCl	N/A	<90 to 60 ml/min OR 10 to <30% decreases from participant’s baseline	<60 to 30 ml/min OR 30 to <50% decreases from participant’s baseline	<30 ml/min OR ≥50% decrease from participant’s baseline or dialysis needed.

**Table-II:** Frequencies according to gender of Hypertension and Diabetes Mellitus

		Male	Female	*p* value

		N (%)	N (%)	
Hypertensive	Yes	30 (54.5)	16 (53.3)	.915*
	No	25 (45.5)	14 (46.7)	
Diabetes Mellitus	Yes	31 (56.4)	14 (46.7)	.392*
	No	24 (43.6)	16 (53.3)
Age ±SD		52.13 ±11.66	50.27 ±16.26	.540**

Significance Level p ≤.05,

* χ^2^ Test,

** Student's T-Test.

**Table-III:** Mean ± SD of Creatinine Clearance & ALT on Days 0, 3, 7 &14

	CrCl^µ^	ALT

	Mean ±SD*	Mean ±SD*
Day 0	126.39 ±51.64	39.91 ±26.89
Day 3	111.97 ±54.99	54.45 ±57.53
Day 7	118.21 ±40.25	46.65 ±45.29
Day 14	134.89 ±59.99	36.74 ±24.97

* Standard Deviation,

µ Creatinine clearance.

Out of 85 patients, Remdesivir was stopped in 3 (3.5%) patients. Among these three patients Remdesivir was stopped in one male patient because of decrease in CrCl to <30 ml/min. In the remaining two patients Remdesivir was stopped on day three due to increase in the ALT levels. Follow up of three patients in whom Remdesivir was stopped due to decrease in CrCl (Patient one) & increased ALT (Patients two & three) on Day three is given in Table-IV.

**Table-IV:** Follow up of 3 patients in whom Remdesivir stopped due to increase in CrCl (Patient 1) & ALT (Patient 2 & 3) on Day 3.

	Parameter	Day 0	Day 3	Day 7	Day 14
Patient 1	CrCl^µ^	88.5	29.5	40.3	55.3
Patient 2	Bilirubin (mg/dl)	0.5	0.7	0.7	0.6
	ALT* (IU/L)	100.0	343.0	280.0	62.0
	Alk Phos	125.0	127.0	122.0	100.0
Patient 3	Bilirubin(mg/dl)	0.5	0.9	0.7	0.4
	ALT (IU/L)	67.0	354.0	247.0	54.0
	Alk Phos**	135.0	194.0	132.0	128.0

µ Creatinine clearance,

* Alanine Transaminase,

** Alkaline Phosphatase.

There was no significant gender difference in patients who developed derangement in hepatic and renal function while on Remdesivir. [χ^2^ (df=1, N=85) = 0.005; *p=0.942*]. There was no significant difference in age in patients who developed derangement in hepatic and renal function while on Remdesivir. t (83) = 1.080, *p* = 0.28.

In one-way ANOVA, there was significant difference in ALT on days three and seven between patients in whom Remdesivir was stopped and not stopped. Day three [F (1, 83) = 67.46, p<0.001)]. Day three [(F (1,83) =48.56, p <0.001)].

## DISCUSSION

Our study demonstrated safety of Remdesivir on liver and kidney function. Majority of patients in our study who were hospitalized and received maximum five days of Remdesivir, did not develop significant hepatotoxicity or nephrotoxicity. Only three out of eighty-five (3.53%) patients developed drug dependent adverse effects to the extent of stopping the drug. Out of these three, only one patient developed significant derangement of creatinine clearance to less than 30 ml/min, and two patients developed significant elevation in ALT to almost 10 times upper limit of normal, necessitating stoppage of Remdesivir on day 3, with gradual improvement in renal and liver functions after stopping the drug.

Mild derangement of liver enzymes, bilirubin and creatinine clearance due to Remdesivir, was noted in some case studies but data regarding severe hepatic and renal function derangement by this drug causing stopping it, is scarce.^13^ It has been documented that abnormalities in liver functions correlates with severity of Covid infection.^14^ Covid infection also had impact on eye and GI hemorrhage and its management.^15^,^16^ Very few studies demonstrated the safety of Remdesivir in hospitalized SARS-CoV-2 patients on renal and hepatic functions. Although SARS-CoV-2 infection can cause aminotransferase elevation, patient 2 had raised ALT levels upto 2.7 times prior to initiating Remdesivir and patient 3 had ALT levels 1.8 times prior to initiating Remdesivir. Their ALT levels increased to more than 9 times after Remdesivir, suggesting a direct role of Remdesivir in hepatocellular toxicity. van Laar SA et al. in their study on 103 hospitalized patients, on oxygen who received five days treatment of Remdesivir, revealed 11% of the patients had a decline in estimated glomerular filtration rate >10 mL/min/1.73m2, 25% had raised ALT and 35% had raised AST levels from their baseline values.^17^ However similar to our study, severe derangements were less, as six out of 103 (5.82%) patients developed eGFR less than 30ml/min and 5/103 (4.85%) developed >5 times ULN of ALT and AST.^17^

World health organization in its Database on 439 individual case reports, highlighted suspected adverse drug events due to Remdesivir and reported elevation in liver enzymes (32.1%), renal injury (14.4%) and increase serum creatinine (11.2%) of patients.^18^ They also reported majority of drug related adverse effects were seen in Americans (67.7%) and mostly in males > 45 years of age,^18^ but in our study we did not find significant age or gender difference in patients who developed derangement in hepatic and renal function while on Remdesivir.

Pettit NN et al. in their comparative study at academic medical center in Chicago, Illinois, gave intravenous solution of Remdesivir.^19^ Out of 137 patients whom they gave Remdesivir, 20 (14.8%) patients already had severe renal impairment as 15 patients had CrCl <30 mL/min and five patients had ESRD that required intermittent hemodialysis, where they concluded that Remdesivir related toxic effects in patients with SARS-COV-2 and severe renal impairment were same as in SARS-COV-2 patients without severe renal impairment as only 4/20 (20%) patients had further serum Cr elevations following Remdesivir, of which three of four patients were already suffering from AKI, before the initiation of RDV.^19^

A study in Pakistan had earlier showd that RDV did not show any difference in in-hospital mortality in Covid19 patients,, more patients had severe ARDS in the RDV group The authors also reported that the length of stay was longer in patients receiving Remdesivir therapy. 20

One of the major strengths of our study is its study design being prospective cohort so provides clarity of the temporal sequence and the selection bias is minimum. We assessed clinical safety of Remdesivir and evaluated the actual clinical need of stopping treatment from severe adverse effect versus theoretical knowledge of Remdesivir adverse effects. There is very limited data available in Pakistan and worldwide on this clinical assessment in cohort of relatively large number of patients.

### Limitations:

We excluded patients with ALT or AST > 5 times the ULN prior starting Remdesivir, known HBV, HDV & HCV or any known liver disease, eGFR < 30 ml/min so more studies with larger sample size would be beneficial to evaluate the effect of Remdesivir in these group of patients in more detail. Another limitation was that this was a single center study and patients were not followed later.

## CONCLUSION

Majority of our patients did not develop any significant liver of renal adverse effects due to Remdesivir. Only three patients developed adverse effects necessitating the stoppage of Remdesivir, however these were reversible on stopping the drug. Therefore, Remdesivir is a relatively safe drug and well tolerated in SARS-CoV-2 patients.

### Authors’ Contribution:

**RS, TR, BFZ:** Substantial contributions to conception and design, or acquisition of data, or analysis and interpretation of data.

**RS, FSA:** Drafting the article or revising it critically for important intellectual content.

**BFZ:** Final approval of the version to be published.

**TR, FSA:** Statistical Analysis.

**All Authors:** Agree to be accountable for all aspects of the work in ensuring that questions related to the accuracy or integrity of any part of the work are appropriately investigated and resolved.
